# Comparison of Serum Sodium Levels Following Intravenous Administration of Isotonic and Hypotonic Solutions in Young Children: A Randomized Controlled Trial

**DOI:** 10.3390/pediatric17060122

**Published:** 2025-11-06

**Authors:** Nisara Chongcharoen, Yupaporn Amornchaichareonsuk, Suwanna Pornrattanarungsi, Ornatcha Sirimongkolchaiyakul

**Affiliations:** 1 Department of Pediatrics, Faculty of Medicine, Vajira Hospital, Navamindradhiraj University, Bangkok 10300, Thailand; nisara_maimy@hotmail.com; 2 Division of Nephrology, Department of Pediatrics, Faculty of Medicine, Vajira Hospital, Navamindradhiraj University, 681 Samsen Rd., Dusit, Bangkok 10300, Thailand; yupaporn.a@nmu.ac.th; 3 Division of Cardiology, Department of Pediatrics, Faculty of Medicine, Vajira Hospital, Navamindradhiraj University, Bangkok 10300, Thailand; suwanna.p@nmu.ac.th

**Keywords:** hypotonic solutions, isotonic solutions, hyponatremia, metabolic acidosis, children

## Abstract

Objectives: This study evaluated changes in serum sodium (S Na) 24 h after the administration of isotonic versus hypotonic intravenous fluids (IVFs) and the incidences of dysnatremia and hyperchloremic metabolic acidosis. Methods: This double-blind, randomized controlled trial involved children aged 3 months to 5 years who were admitted to a general ward between November 2020 and September 2022 and required IVF. We randomly assigned patients (1:1) to receive either an isotonic solution (D_5_0.9%NaCl) or hypotonic solution (D_5_0.45%NaCl). Serum electrolyte and venous blood gas levels were obtained at the time of IVF administration and 24 and 48 h after IVF administration. During this study, all participants were monitored for vital signs, body weight, fluid intake and output, and clinical symptoms of dysnatremia. Results: Totals of 69 and 68 patients received isotonic and hypotonic solutions, respectively. The mean age was 1.95 ± 1.25 years in the isotonic group and 1.91 ± 1.32 years in the hypotonic group. The initial degrees of dehydration and biochemical indicators were not different. The change in serum sodium level at 24 h was 2.97 (2.32–3.62) mmol/L in the isotonic group and 2.19 (1.54–2.84) mmol/L in the hypotonic group. In both groups, no significant hyponatremia nor hypernatremia occurred. The incidence of hyperchloremic metabolic acidosis was not different between the groups. Neither group showed any complications. Conclusions: Isotonic fluids may be a preferred option for IVFs in pediatric patients under 5 years of age with medical conditions on a general ward, especially within 24 h, due to their potential to better maintain serum sodium levels without increasing the risk of fluid overload or electrolyte complication.

## 1. Introduction

Supportive care using intravenous fluids (IVFs) has been provided to children who were being treated in hospitals and could not take oral intake. During the past decade, evidence has suggested that hypotonic IVF (sodium 30–100 mEq/L) administration increases the risk of hyponatremia, particularly hyponatremic encephalopathy [1,2], which increases the risk of morbidity and mortality. Currently, an isotonic solution is used worldwide for children who are past the newborn period and do not have underlying diseases [3].

However, interesting questions must be answered. Young children are at a high risk of developing hyponatremia from hypotonic fluids [4]. A few studies have undertaken specific analysis in this age group [5,6,7]. Common causes of admission—such as gastroenteritis or bronchiolitis, which are the most common etiologies for admission at our study site—in younger children have a high prevalence of developing hyponatremia compared with older children [8,9]. Some studies have focused specifically on general wards [6,7,10], where most pediatric admissions occur, and on common diseases. Past studies on fluid types have been broad, encompassing both medical and surgical conditions [11,12,13]. Furthermore, young children are at a high risk of developing hypernatremic complications from isotonic solutions, and there are limited significant findings in the previous studies on the newborn period [14].

Moreover, isotonic solutions have a higher chloride component than human plasma and can cause hyperchloremic metabolic acidosis, which is associated with acute kidney injury and gastrointestinal dysfunction in critically ill children [15]. Past studies that examined these effects focused on critically ill pediatric populations within intensive care units (ICUs); as such, there is a paucity of data regarding the incidence and clinical significance of electrolyte and acid–base imbalances in non-ICU children receiving isotonic fluids [4], and specific studies that involved young children are scarce. ICU patients experience severe diseases, complex complications, and stressful events that affect the electrolyte balance control in the body compared with patients admitted to the general ward. Moreover, young children have a physiological profile that is distinct from older children, including a greater proportion of total body water and a different ratio of extracellular to intracellular fluid [16]. This age group has immature renal function. Young children are potentially more vulnerable to electrolyte disturbances. Therefore, this study was conducted to evaluate the changes in mean serum sodium levels 24 h after the administration of isotonic versus (vs.) hypotonic solutions in young children who were admitted to a non-ICU ward. The secondary objectives included the incidences of hyponatremia, hypernatremia, and hyperchloremic metabolic acidosis and changes in the mean serum sodium levels 48 h after IVF administration.

## 2. Materials and Methods

A randomized double-blind controlled trial was conducted at the general inpatient ward of Vajira Hospital, Bangkok, Thailand, between November 2020 and September 2022. Children aged 3 months to 5 years who required IVF treatment with initial serum sodium levels of 130–140 mmol/L were enrolled in this study. The exclusion criteria were as follows: neurosurgical disorders, congenital or acquired heart disease, hepatic disease, cancer, renal dysfunction, adrenal insufficiency, diabetes insipidus, voluminous watery diarrhea, severe burn, shock, and preexisting metabolic or electrolyte disorders. This study was conducted in accordance with the Declaration of Helsinki and approved by the Institutional Review Board (COA 100/2563). The trial was registered with the Thai Clinical Trials Registry under the number TCTR20201212004 on 12 December 2020. Note that this study was originally placed in this retrospective registry because of miscommunication between the research team; the research team was promptly registered correctly upon the detection of the error.

### 2.1. Intervention

The randomization sequence involved a balanced block of four computer-generated methods supervised by a person who was not directly involved in this study, and the code was opened after the parents were allowed to enroll their children in this study. Patients (1:1) were allocated to receive isotonic solution (0.9% NaCl in 5% dextrose; sodium 154 mmol/L) or hypotonic solution (0.45% NaCl in 5% dextrose; sodium 77 mmol/L). The randomization code was kept in a secure location and was not accessible to the research team. The code was only to be unblinded in the case of a serious adverse event, such as a severe electrolyte disturbance requiring immediate, specific treatment. This process ensured the integrity of this study by preventing bias that influenced the patient care or outcome assessment. The fluid types were masked by a person not involved in this study using opaque paper that was identical in appearance and labeled as A or B. This process enabled us to conceal the identities of the IVFs from the patient, family, nurses, physicians, and research team. Informed consent was obtained from the parents before the patients participated in this study.

The rate of IVF administration was calculated according to the Holliday–Segar formula, and the level of dehydration was managed by a treating physician who was not part of the research team. Serum electrolyte and venous blood gas levels were measured before or within 1 h of the initial administration of IVF and at 24 and 48 h if the patient continued to receive the IVF. During this study, vital signs were monitored every 4 h, fluid intake and output were recorded every 8 h, and body weight was assessed every day. Moreover, the research team closely monitored the complications of the IVF treatment, such as volume overload and electrolyte imbalance. Any patient that developed a serious clinical electrolyte disturbance, for example, alteration of consciousness or seizure, stopped receiving the IVF and serum electrolyte levels were measured immediately.

### 2.2. Outcomes

The primary outcome was the change in serum sodium levels 24 h after the administration of isotonic vs. hypotonic solutions. The secondary outcome was the incidence of hyponatremia (S Na < 135 mmol/L), hypernatremia (S Na > 145 mmol/L), and hyperchloremic metabolic acidosis (serum chloride ≥ 110 mmol/L [17,18] and serum bicarbonate < 20 mmol/L or venous pH < 7.35 [19]). Moreover, the difference in serum sodium levels after 48 h of the initial IVF administration between isotonic and hypotonic solutions was assessed.

### 2.3. Statistical Analysis

To detect changes in the serum sodium levels 24 h after receiving different IVFs, 63 patients per group were required, which was calculated based on a previous study [6] with a power of 80% and a two-sided alpha level of 5%. Assuming a 10% loss to follow-up, the sample size was increased to 70 patients in each group.

Analysis was performed using the intention-to-treat method. Continuous data are expressed as means ± standard deviations (SDs) or medians (interquartile ranges [IQRs]), whereas categorical data are expressed as percentages. Depending on the data’s normality, either the Mann–Whitney U test or independent t-test was performed to examine the group differences. Categorical data were analyzed using the chi-square test or Fisher’s exact test. To assess the outcomes, the relative risk or mean difference was calculated. Comparison of the outcomes between the isotonic and hypotonic solutions was planned to be performed using generalized linear regression for a binary outcome to estimate the risk ratio. However, we encountered convergence problems during the analysis, which are typical for log binomial regression. The analysis was switched to a modified Poisson regression with a robust error variance [20]. A *p*-value ≤ 0.05 was used to denote statistical significance. All statistical data were analyzed using the Statistical Package for the Social Sciences (version 23.0; IBM Corp., New York, USA).

## 3. Results

**Figure 1:**
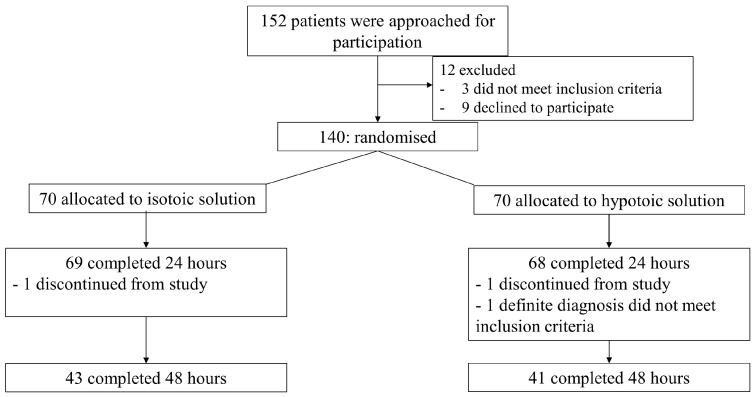
Flow diagram of patients assigned to the study.

Of the 152 potentially eligible patients identified in the pediatric ward, 12 patients were excluded because 3 patients did not meet the inclusion criteria because of their initial serum sodium level not being 130–140 mmol/L and 9 patients declined to participate. Finally, 140 patients were randomized. Seventy patients were allocated to the isotonic group; the remaining seventy patients were allocated to the hypotonic group. The isotonic group consisted of 69 subjects because 1 patient withdrew from the trial. One patient in the hypotonic group discontinued this study before 24 h, and one patient was diagnosed with Kawasaki disease; thus, the hypotonic group had sixty-eight patients. IVF administration was discontinued because of clinical improvement and increased oral acceptability. Finally, the isotonic group had 43 patients and the hypotonic group had 41 patients who had completely received IVF at 48 h (Figure 1).

Table 1 shows the baseline characteristics of the patients. The mean ages in the isotonic and hypotonic groups were 1.95 ± 1.25 and 1.91 ± 1.32 years, respectively, where no significant difference in age was observed between the two groups. The baseline characteristics were similar between both groups, including sex, body weight, diagnosis, initial temperature, and degree of dehydration. Of the patients in the isotonic group, 27.1% were unable to eat or drink by mouth on the first day due to respiratory distress or seizures compared with 22.8% in the hypotonic group. This difference was not statistically significant (*p*-value = 0.55). The mean serum sodium, chloride, bicarbonate, and pH levels from venous blood gas were not different between the isotonic and hypotonic groups. Furthermore, the IVF volumes and oral intakes were similar in both groups. However, the amounts of sodium received differed according to the study protocol (isotonic group > hypotonic group) (Table 2).

### 3.1. Primary Outcome

The primary outcome was the change in serum sodium levels from baseline to 24 h. The isotonic group showed a change of 2.97 mmol/L (IQR, 2.32–3.62), while the hypotonic group’s change was 2.19 mmol/L (IQR, 1.54–2.84). These changes were not statistically different (mean difference, 0.78; 95% confidence interval [CI], −0.14 to 1.70; *p*-value = 0.09). Table 3 shows the outcomes.

### 3.2. Secondary Outcomes

The incidence of hyponatremia 24 h after the IVF administration was higher in the hypotonic group than in the isotonic group (13.2% vs. 7.3%); however, the difference was not statistically significant (relative risk, 0.55; 95% CI, 0.19 to 1.56; *p*-value = 0.25). Neither severe hyponatremia (S Na < 125 mmol/L) nor hypernatremia occurred in any participant after receiving IVF for 24 h. At 24 h, the incidence of hyperchloremic metabolic acidosis was 8.7% in the isotonic group and 2.9% in the hypotonic group. No significant difference was observed (relative risk, 2.96; 95% CI, 0.61 to 14.22; *p*-value = 0.17). At 48 h from baseline, there was no change in the serum sodium levels: 2.76 mmol/L (2.00–3.51 mmol/L) in the isotonic group vs. 2.41 mmol/L (1.61–3.22 mmol/L) in the hypotonic group (mean difference, 0.35; 95% CI, −0.76 to 1.45; *p*-value = 0.53).

### 3.3. Side Effects

There were no changes in body weight 24 and 48 h after the initial treatment between the two groups (24 h: mean difference, 0.01; 95% CI, −0.77 to 0.78; *p*-value = 0.98) (48 h: mean difference, −0.27; 95% CI, −1.36 to 0.83; *p*-value = 0.62). Moreover, hypertension did not develop in either group (Table 4), indicating no notable fluid overload in either group. During the study period, the patients did not have any edema, alteration of consciousness, or seizure from electrolyte imbalance or volume overload. The hospital stay duration was the same in both groups (3.35 ± 1.40 days vs. 3.34 ± 1.62 days in the isotonic vs. hypotonic groups, respectively; mean difference, 0.01; 95% CI, −0.50 to 0.52; *p*-value = 0.97).

## 4. Discussion

A double-blind randomized controlled trial was conducted to compare isotonic (0.9% NaCl in 5% dextrose) and hypotonic (0.45% NaCl in 5% dextrose) solutions. The changes in the mean serum sodium level 24 h after receiving IVF were similar between the young non-ICU patients, independent of their medical condition. This finding is different from that of Ramanathan et al. [10], who only studied patients with severe pneumonia and compared 0.9% NaCl with 0.18% NaCl, finding that the mean serum sodium significantly changed at 0 to 24 h but not significantly at 12 to 24 h. Kumar et al. [6] found a significant change in serum sodium levels 24 h after baseline in patients with medical problems; the most common diagnosis was respiratory diseases in the isotonic group (52.4%) and hypotonic group (52.4%) and the second diagnosis was central nervous system diseases in the isotonic (23.8%) and hypotonic (22.6%) groups. As the diagnosis had an influence on the hyponatremia, the frequency of diagnosis was different from our study. A meta-analysis and systematic review showed that the hypotonic solution only led to lower serum sodium levels after less than 24 h when compared with the isotonic solution [21]. A meta-analysis and systematic review [21] included differences in the cause of admission and fluid types, in contrast with the current study (Table 5).

Our findings show that the incidence of hyponatremia at 24 h in the hypotonic group (13.2%) was higher than that in the isotonic group (7.3%); however, the difference was not statistically significant (*p*-value = 0.25). Despite no statistically significant difference in the incidence of hyponatremia between the isotonic and hypotonic groups, the fact that the incidence of this adverse event was not higher in our isotonic group and trended lower may provide evidence for the clinical appropriateness of using isotonic fluids in this population. Our findings, while not statistically significant, align with Kumar et al. [6] and Friedman et al. [22], who revealed the same finding in children who were acutely ill in general wards. Fernández-Sarmiento et al. [23] found the same result in patients admitted to pediatric ICUs. However, a systematic review and meta-analysis [21,24] of previous studies showed a significantly higher incidence of hyponatremia in patients that received a hypotonic treatment vs. an isotonic treatment, which included the ICU setting, surgical conditions, neonates, and varying types of fluids (0.18% NaCl, 0.3% NaCl, 0.45% NaCl) because non-osmotic stimuli to antidiuretic hormone secretion are triggered by pain, stress, nausea, and operations; moreover, surgical patients also had fluid leaking, which worsened their hyponatremia. Although the IVF rates in the earlier studies and our analysis were different, Wang et al. [25] discovered that the type of IVF—not its rate—affected the hyponatremia risk. The safer period regarding the hyponatremia risk from isotonic solution is 72 h following therapy; this study monitored serum sodium levels at 24 and 48 h, which can be applied in clinical practice. This study supported a previous result [26] showing that the incidence of hypernatremia was comparable across groups. Hypernatremia is of concern when using an isotonic solution, but this event did not occur in the isotonic group in this study. This underscores the importance of a clinically driven approach that prioritizes patient safety over statistical variations.

The important point against using isotonic solutions was inducing hyperchloremic metabolic acidosis because of the higher chloride component than that in human plasma. Our study demonstrated an incidence of 8.7% 24 h after the isotonic fluid treatment, which was consistent with that of hypotonic fluid. A similar result was obtained from a previous randomized controlled trial conducted in general wards and ICUs [12]. However, a retrospective cohort study in ICUs found that isotonic solutions increased the hyperchloremic metabolic acidosis risk, which was different in this study for patients in the general ward. Although one-third of the patients received isotonic solution (0.9% NaCl) at 10 mL/kg in 1 h because of moderate dehydration, this bolus volume was insufficient to generate hyperchloremic metabolic acidosis. The other adverse effects of isotonic solutions were hypertension and increased body weight, which were not found in this study because the volume intake was comparable between the two groups, and hypernatremia did not occur in the isotonic group. Regarding the outcome, the length of stay was the same between the two groups, in agreement with Robles et al. [13] and Hasim et al. [24].

### Limitations

This study’s strength was that it was a double-blind randomized controlled trial in a particular age group of children who had nonsurgical issues and were admitted to the general ward, which is practical for pediatricians; however, our findings might not be generalizable to other children. While the difference in serum sodium between the isotonic and hypotonic groups was found to be statistically insignificant, we recognize a critical limitation regarding its clinical interpretation. Future study must correlate statistically significant differences in serum sodium with these definitive clinical endpoints to establish true clinical superiority. Because some young patients were breastfeeding and had uncontrolled urination, determining the precise volume intake and output was very challenging. This study did not have detailed data on the sodium contents of the milks and foods consumed by the participants. Future research should consider including this information to provide a more comprehensive analysis. Furthermore, to determine the incidence of significant hyperchloremic metabolic acidosis, we needed a larger sample size and to enroll critically ill patients in the ICU.

## 5. Conclusions

Isotonic solutions may be a suitable option for maintaining hydration in pediatric patients under 5 years of age who have medical issues in a general ward. Isotonic solution helps with maintaining the serum sodium level and does not significantly increase the electrolyte disturbance or clinical complications. However, the practical point of care for managing IVF involves clinical adjustment and monitoring. Future studies should focus on long-duration outcomes and the potential for complications in more critically ill young pediatric populations, where the fluid balance is more sensitive.

## Figures and Tables

**Table 1:** Baseline characteristic in isotonic and hypotonic groups.

Parameters	Isotonic Group (n = 69) (n, %)	Hypotonic Group (n = 68) (n, %)	*p*-Values
**Age (years)** ^†^	1.95 ± 1.25	1.91 ± 1.32	0.86
**Sex**			
Male	45 (65.2)	40 (58.8)	0.44
**Diagnosis**			0.90
Acute gastroenteritis	36 (52.2)	35 (51.5)	
Pneumonia	10 (14.5)	9 (13.2)	
Upper respiratory tract infection	3 (4.3)	6 (8.8)	
Urinary tract infection	5 (7.3)	4 (5.9)	
Others	15 (21.7)	14 (20.6)	
**Body weight (kg)** ^†^	11.65 ± 4.20	11.90 ± 4.61	0.74
**Initial temperature (°C)** ^†^	37.57 ± 1.00	37.60 ± 0.96	0.84
**Nothing per mouth at first 24 h**	19 (27.1)	16 (22.8)	0.55
**Degree of dehydration**			0.73
No	21 (30.4)	25 (36.8)	
Mild	27 (39.1)	24 (35.3)	
Moderate	21 (30.4)	19 (27.9)	
**Initial serum sodium (mmol/L)** ^†^	135.06 ± 2.36	135.13 ± 2.64	0.86
**Initial serum chloride (mmol/L)** ^†^	101.32 ± 3.25	101.54 ± 3.80	0.71
**Initial serum bicarbonate (mmol/L)** ^†^	18.62 ± 3.48	18.62 ± 4.17	0.99
**Initial venous blood gas pH (corrected)** ^†^	7.40 ± 0.05	7.40 ± 0.05	0.92
**Initial base excess**	−6.14 ± 3.61	−6.29 ± 4.52	0.82
**Initial serum potassium (mmol/L)** ^†^	4.14 ± 0.44	4.21 ± 0.40	0.29
**BUN (mg/dL)** ^†^	11.68 ± 4.76	10.81 ± 3.92	0.27
**Serum Cr (mg/dL)** ^†^	0.33 ± 0.08	0.33 ± 0.09	0.93

BUN: blood urea nitrogen; Cr: creatinine; kg: kilogram; mg/dL: milligram/deciliter; mmol/L: millimoles/liter; n: number of participants. ^†^ mean ± SD.

**Table 2:** Volume and sodium administration in isotonic and hypotonic groups.

Parameters	Isotonic Group (n = 69)	Hypotonic Group (n = 68)	*p*-Values
**IVF volume during study (mL/kg/day)** ^†^			
At 24 h	112.29 ± 29.68	105.39 ± 30.57	0.18
At 48 h	80.03 ± 26.98	79.66 ± 28.16	0.95
**Oral fluid intake (mL/kg/day)** ^†^			
At 24 h	65.70 ± 39.16	60.95 ± 38.18	0.50
At 48 h	77.70 ± 57.76	85.70 ± 58.46	0.56
**Sodium received (mmol/L/kg/day)** ^†^			
At 24 h	18.50 ± 5.42	8.85 ± 2.68	<0.001
At 48 h	12.71 ± 4.39	6.66 ± 2.15	<0.001

IVF: intravenous fluid; h: hour; kg: kilogram; mL: milliliter; mmol/L: millimoles/liter; n: number of participants. ^†^ mean ± SD.

**Table 3:** Primary and secondary outcomes in electrolyte parameters.

Outcomes	Isotonic Group (n = 69) (n, %)	Hypotonic Group (n = 68) (n, %)	Relative Risk ^a^/Mean Difference ^b^ (95%CI)	*p*-Values
**Change in S Na (mmol/L)** ^‡^				
At 24 h	2.97 (2.32–3.62)	2.19 (1.54–2.84)	0.78 (−0.14–1.70)	0.09
At 48 h	2.76 (2.00–3.51)	2.41 (1.61–3.22)	0.35 (−0.76–1.45)	0.53
**Incidence of hyponatremia** **(S Na < 135 mmol/L)**				
At 24 h	5 (7.3)	9 (13.2)	0.55 (0.19–1.56)	0.25
At 48 h	6 (8.7)	3 (4.4)	1.97 (0.51–7.60)	0.32
**Incidence of severe hyponatremia (S Na < 125 mmol/L) at 24 h**	0 (0)	0 (0)	–	NA
**Incidence of hypernatremia (S Na >145 mmol/L) at 24 h**	0 (0)	0 (0)	–	NA
**Incidence of hyperchloremia**				
At 24 h	14 (20.3)	7 (10.3)	1.97 (0.85–4.59)	0.11
At 48 h	8 (11.6)	3 (4.4)	2.63 (0.72–9.53)	0.14
**Incidence of metabolic acidosis**				
At 24 h	28 (40.6)	28 (41.2)	0.99 (0.66–1.48)	0.94
At 48 h	12 (17.4)	15 (22.1)	0.79 (0.40–1.56)	0.49
**Incidence of HCMA**				
At 24 h	6 (8.7)	2 (2.9)	2.96 (0.61–14.22)	0.17
At 48 h	2 (2.9)	1 (1.5)	1.97 (0.18–21.42)	0.57

CI: confidence interval; h: hour; HCMA: hyperchloremic metabolic acidosis; mmol/L: millimoles/liter; n: number of participants; NA: not applicable; S Na: serum sodium. Isotonic group n = 43 and hypotonic group n = 41 at 48 h. ^‡^ median (IQR); ^a^: relative risks were represented in categorical variables; ^b^: mean differences were represented in continuous variables.

**Table 4:** Side effects of intravenous fluids administration.

Parameters	Isotonic Group (n = 70) (n, %)	Hypotonic Group (n = 70) (n, %)	Relative Risk ^a^/Mean Difference ^b^ (95%CI)	*p*-Values
**Hypertension**	0 (0)	0 (0)	–	NA
**Change in BW (kg)** ^‡^				
At 24 h	0.33 (−0.22–0.89)	0.33 (−0.24–0.89)	0.01 (−0.77–0.78)	0.98
At 48 h	0.01 (−0.71–0.73)	0.28 (−0.48–1.04)	−0.27 (−1.36–0.83)	0.62
**Length of stay**	3.35 ± 1.40	3.34 ± 1.62	0.01 (−0.50–0.52)	0.97

BW: body weight; CI: confidence interval; h: hour; kg: kilogram; n: number of participants; NA: not applicable. Isotonic group n = 43, hypotonic group n = 41 at 48 h. ^‡^ median (IQR); ^a^: relative risks were represented in categorical variables; ^b^: mean differences were represented in continuous variables.

**Table 5:** Comparison of primary key finding (changed serum sodium) with previous studies.

Study	Patient Population	Condition	Solution	Follow-Up Duration	Changed Serum Sodium
Current study; RCT	Age 3 months–5 years	Medical, general ward	0.9% NaCl in 5% dextrose vs. 0.45% NaCl in 5% dextrose	24 and 48 h	At 24 h; isotonic group 2.97 (IQR 2.32–3.62) vs. hypotonic group 2.19 (IQR 1.54–2.84): MD = 0.78; *p*-value = 0.09
Ramanathan et al. [10]; RCT	Age 2 months–5 years	Severe pneumonia, general ward	0.9% NaCl in 5% dextrose vs. 0.18% saline in 5% dextrose	6, 12 and 24 h	At 0–24 h; isotonic group 2.8 ± 6.8 vs. hypotonic group −3 ± 6.3: MD = 5.8; *p*-value = <0.001 at 12–24 h; isotonic group 1.3 ± 4.2 vs. hypotonic group 1.4 ± 4.9: MD = −0.04; *p*-value = 0.963
Kumar et al. [6]; RCT	Age 3 months–5 years	General ward	0.9% NaCl in 5% dextrose vs. 0.45% NaCl in 5% dextrose	12 and 24 h	At 24 h from baseline; isotonic group 2.14 ± 4.7 vs. hypotonic group –0.24 ± 4.8: MD = 2.38; *p*-value = 0.002
Amer et al. [21]; systematic review and meta-analysis	Age 1 day–18 years	Medical and surgical conditions, general ward and ICUs	0.9% NaCl, Ringer’s lactate, or Hartmann’s solution vs. 0.18% NaCl, 0.3% NaCl, or 0.45% NaCl	≤24 and >24 h	Hypotonic group had lower serum sodium level compared with isotonic group at ≤24 h (MD = −2.36, *p*-value = < 0.00001)

h: hour; ICU: intensive care unit; IQR: interquartile range; MD: mean difference; RCT: randomized control trial; vs.: versus.

## Data Availability

The data presented in this study are available upon request from the corresponding author due to confidentiality reasons.

## Abbreviations

BUN: blood urea nitrogen; BW: body weight; CI: confidence interval; Cl: chloride; Cr: creatinine; h: hour; HCMA: hyperchloremic metabolic acidosis; HCO3: bicarbonate; ICU: intensive care unit; IQRs: interquartile ranges; IVFs: intravenous fluids; kg: kilogram; mL: milliliter; mg/dL: milligram/deciliter; mmol/L: millimoles/liter; n: number of participants; NA: not applicable; Na: sodium; SDs: standard deviations; vs.: versus
